# Rodent Ulcer

**Published:** 1905-01-14

**Authors:** 


### RODENT ULCER.

IN a lecture given at St. George's Hospital, and reported in the *Clinical Journal*, Mr. Sheild called attention to some noteworthy points in connection with the treatment of rodent ulcer, and especially to the necessity for early operation. He advises that any wart, mole, or pigment spot on the face of a person over 40 years of age, which increases in size, which itches, "burns," or becomes sore or scabbed, should be excised at once. The practice of applying inefficient caustics to such formations is very injurious. Such imperfect treatment only aggravates the disease. The fear of making a scar causes a number of operations to be incomplete and unsatisfactory; in fact they are seldom carried out with sufficient freedom. A great help to getting immediate union in these cases, and so lessening the resultant scar, is to thoroughly detach the surrounding skin from subjacent parts by introducing a tenotome around the margins of the incisions. Mr. Sheild deprecates the use of local anæsthetics in removing rodent ulcers from the face. The injection of cocaine, he states, is dangerous, whilst eucaine is not sufficient to ensure that absolute passiveness that is essential if the best results are to be obtained.

The operation is liable to be hurried, and the union of the wound margins to be badly executed, so that an unsightly scar, or even serious deformity, especially if the wound be near the eyelids, may be left. *X*-rays may be used if excision is impossible or is refused, but it is a tedious and expensive treatment, not free from danger, and not always beneficial. In all cases, and whatever treatment is employed, caution must be used in giving a prognosis. Local recurrence is the rule in rodent ulcer whatever measures are employed, but free and wide removal offers the best chance of a long period of freedom.

